# Analysis in Choroidal Thickness in Patients with Graves' Ophthalmopathy Using Spectral-Domain Optical Coherence Tomography

**DOI:** 10.1155/2018/3529395

**Published:** 2018-12-23

**Authors:** Nan Yu, Yadi Zhang, Lei Kang, Ying Gao, Junqing Zhang, Yuan Wu

**Affiliations:** ^1^Department of Endocrinology, First Hospital, Peking University, Beijing, China; ^2^Department of Ophthalmology, First Hospital, Peking University, Beijing, China

## Abstract

**Objectives:**

The objective of the study is to observe changes in choroidal thickness (CT) in patients with Graves' ophthalmopathy using spectral-domain optical coherence tomography (SD-OCT).

**Methods:**

The right eyes of 36 patients (27 females and 9 males) with Graves' ophthalmopathy (GO) and those of 36 age-, gender-, and diopter-level-matched healthy participants were evaluated. The patients' data were obtained within 3 months after the onset of Graves' disease (GD). Thyroid hormone levels and thyroid-stimulating hormone receptor antibody (TRAb) levels were measured, and the degree of exophthalmos was measured in all patients. Activity is measured by the clinical activity score (CAS). A horizontal scan centered on the fovea was performed in all participants. Five points of choroidal thickness were measured at the fovea (SFCT) and at 1500 *μ*m nasal (N1500), 3000 *μ*m nasal (N3000), 1500 *μ*m temporal (T1500), and 3000 *μ*m temporal (T3000) to the fovea.

**Results:**

The CT measurements obtained were (mean ± SD) 313.47 ± 100.32 *μ*m, 279.22 ± 85.80 *μ*m, 214.64 ± 75.52 *μ*m, 313.19 ± 80.36 *μ*m, and 298.14 ± 82.75 *μ*m in patients with GO and were 256.33 ± 50.18 *μ*m, 223.14 ± 59.61 *μ*m, 176.69 ± 60.66 *μ*m, 250.92 ± 52.184 *μ*m, and 239.47 ± 60.35 *μ*m in the control group at the foveal, N1500, N3000, T1500, and T3000 measurement points, respectively. The CT in GO patients was significantly increased at all the points compared with the control group (*P* < 0.05). There was no relationship between the CT and CAS, the degree of exophthalmos, triiodothyronine (T3), tetraiodothyronine (T4), thyroid-stimulating hormone (TSH), or TRAb levels in GO.

**Conclusions:**

CT was found to be increased in GO patients and had poor relationship with CAS, exophthalmos, and thyroid function tests.

## 1. Introduction

Graves' disease (GD) is a common kind of autoimmune thyroid disease. It is characterized by diffuse goiter and thyrotoxicosis and may be accompanied by an infiltrative ophthalmopathy and occasionally by an infiltrative dermopathy. A remarkable complication of GD is ocular injures, also known as Graves' ophthalmopathy (GO). The pathogenesis of GO involves massive cellular and fluid infiltration of the soft tissues of bony orbit and extraocular muscles; thus, retracted upper eyelids, protuberant eyeballs, and limited eye movements are found in GO patients. There is also an inflammatory stage at the onset of GO, and injection or chemosis of the ocular surface, corneal fluorescein staining, and meibomian gland dysfunction may occur during this stage.

In hyperthyroidism patients, the cardiac output, systolic blood pressure, and heart rate may increase, and the systemic vascular resistance and diastolic blood pressure may decrease [1]. The hemodynamics of several organs may change including those of the eye [2, 3]. Previous studies have mentioned that active GO patients have increased retinal blood flow. Several techniques, such as color Doppler imaging, ocular blood flow tonography, and oculodynamometry, can provide more accurate information about ocular blood flow [4, 5].

Studies on choroidal thickness (CT) have become widespread in systemic diseases since it was first reported that measurements could be made in vivo with optical coherence tomography (OCT) [6]. As the vascular membrane of the eye, the choroid provides oxygen and nutrients to the external layers of the retina and the prelaminar portion of the optical nerve. The study of the choroid may promote an understanding of the physiopathology of ocular diseases in which this vascular layer may play an important role and may show that the choroid can serve as an indicator for monitoring changes in various systemic diseases associated with vascular dysfunction.

In this context, we explored changes in choroidal thickness in the eyes of patients with Graves' ophthalmopathy and compared them with those of healthy eyes. An additional objective was to assess the correlation between choroidal thickness, ocular proptosis, and thyroid function testing. There is a lack of similar research in Asian populations. In addition, previous studies did not set a strict control group to match.

## 2. Subjects, Materials, and Methods

A study group including 36 patients diagnosed with GD (27 females and 9 males) at the Department of Endocrinology at Peking University First Hospital during the period between January 2014 and December 2017 and a control group of 36 healthy subjects (36 eyes) examined at the Ophthalmology Department at the same hospital during the same period were included in the analysis. The patients were enrolled within 3 months after the diagnosis of GD was made. The criteria for GD included the following: (1) signs and symptoms of hyperthyroidism, (2) diffuse goiter, (3) elevated triiodothyronine (T3) and tetraiodothyronine (T4) levels and suppressed thyroid-stimulating hormone (TSH) levels, and (4) elevated thyroid-stimulating hormone receptor antibody (TRAb) levels [7]. All of the patients had ocular complications and were also diagnosed with GO, including symptoms of exophthalmos, squinting, eyelid congestion, eyelid edema, conjunctival congestion, and conjunctival edema. We used a clinical activity score (CAS) grading system to evaluate the activity of GO. The items included the following: (1) spontaneous orbital pain, (2) gaze-evoked orbital pain, (3) eyelid swelling that was considered to be due to active GO, (4) eyelid erythema, (5) conjunctival redness that was considered to be due to active GO, (6) chemosis, and (7) caruncle inflammation or plica. A CAS ≥3/7 indicated active GO [8]. Patients who had any other ocular disease (other than refractive errors less than −9.00 DS) or previous surgeries were excluded. The degree of exophthalmos (ocular proptosis) and thyroid function testing results were recorded. The eyes of patients were divided into four levels according to the spherical equivalent: emmetropia level (from +1.00 DS to −0.50 DS), low myopia level (from −0.50 DS to −3.00 DS), moderate myopia level (from −3.00 DS to −6.00 DS), and high myopia level (from −6.00 DS to −9.00 DS).

The control group consisted of age-, gender-, and diopter level-matched healthy individuals without any ocular disease other than refractive errors. Their thyroid function testing results and TRAb level were within the normal range. 36 participants (27 females and 9 males) were enrolled. Every patient was assigned a control subject with the same age, gender, and diopter level (Table 1).

The Ethics Committee of the Research Office of the First Hospital of Peking University approved the study in accordance with the Helsinki declaration and the laws of China. All subjects signed an informed consent form.

### 2.1. Ocular Examination

The right eyes of all the subjects were evaluated. The ophthalmological examination included an anterior pole study with a slit lamp, an intraocular pressure measurement with applanation tonometry, and posterior pole biomicroscopy with a noncontact lens. Refraction was performed with an automatic refractometer, and ocular proptosis was measured with an Hertel exophthalmometry. Optical coherence tomography was performed in all the patients.

**Figure 1:**
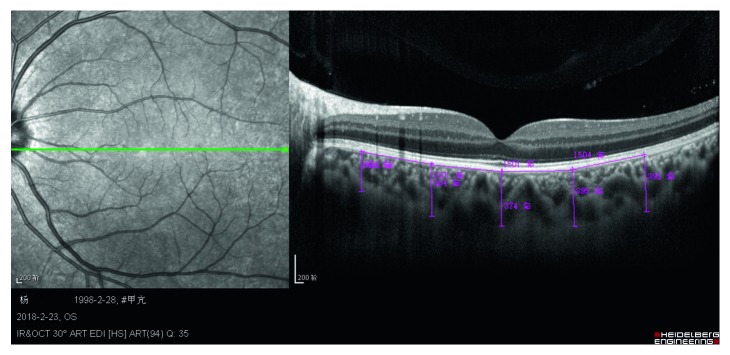
Choroidal thickness in a participant using enhanced depth imaging OCT (from left to right: N3000, N1500, SFCT, T1500, and T3000).

Choroidal thickness was measured with a HD-OCT (length: 870 nm, Spectralis OCT, Heidelberg Engineering, Germany) with an enhanced depth imaging technique (EDI-OCT). A trained technician blinded to the study group performed the scans after the ophthalmological examination. A one-line 6 mm horizontal scan was made with a high definition protocol focused on the fovea. Utilizing the software gauge, five measurements were made perpendicular to the pigment epithelium from the posterior part up to the sclerochoroidal junction. The five measured points included a foveal-centered subfoveal choroidal thickness (SFCT) and four points at the following areas: 1500 *µ*m (N1500) and 3000 *µ*m (N3000) nasal to the fovea and 1500 *µ*m (T1500) and 3000 *µ*m (T3000) temporal to the fovea (Figure 1). The mean of the five measurements was calculated for statistical analysis. The scans with a quality above 6 were selected.

### 2.2. Detection of Thyroid Function

Serum samples from all of the patients were collected and tested. T3, T4, and TSH levels were measured using a chemiluminescent analyzer (ADVIA Centaur, Germany). TRAb levels were measured using another chemiluminescent analyzer (COBAS e601, Germany). Normal ranges are as follows: T3: 0.92–2.79 nmol/L; T4: 58.1–140.6 nmol/L; TSH: 0.35–5.5 *μ*U/mL; TRAb: 0–1.75 IU/L.

### 2.3. Statistical Analyses

The statistical analyses were performed with SPSS Statistics (version 20.0, IBM-SPSS, Chicago, IL, USA). An independent *t*-test was utilized to determine possible significant differences between the GO patients and the matched healthy controls. Univariate analysis was utilized to study the associations between CT, CAS, refractive errors groups, exophthalmometric value, and thyroid function testing. The statistically signification level was under 5% (*P* < 0.05).

## 3. Results

Overall, 36 patients with GO and 36 disease-free volunteers were included in the study, and their eyes were evaluated. All subjects were Han Chinese, and their age ranged from 19 to 76 years (mean ± standard deviation, 39.8 ± 14.5 years). 31 of all subjects were Grave's disease patients without any other systemic disorder. Five of the patients combined with hyperlipidemia, osteoporosis, or well-controlled hypertension. The exophthalmometric value ranged from 12 mm to 30 mm (mean ± standard deviation, 20.7 mm ± 3.8 mm). The CAS ranged from 0 to 7 and 22.2% of the patients had active GO (8/36) when the ocular examinations were performed. The average values of the CT are shown in Table 2. The CT was greater in the GO group than that in the control group. There were statistically significant differences observed at all the points.

A statistical relationship of the four refractive errors level and CT (central and nasal) was found in the control group but not found in the GO group (Table 3).

Possible associations between the mean CT and other factors were studied. There was no statistically significant relationship between CT and CAS, the exophthalmometric value, TRAb, serum T3 levels, and serum T4 levels.

## 4. Discussion

Changes in orbital blood flow have been documented in patients with GO; however, few studies evaluating the CT in Asian population have been published until now. In our study, CT was found to be significantly increased in GO patients compared with age-, gender-, and diopter level-matched healthy individuals. There was no statistically significant relationship between CT and CAS, the exophthalmometric value, TRAb, serum T3, and T4 levels.

Graves' disease, which is also known as toxic diffuse goiter, is a common autoimmune disorder that may lead to systemic changes in many organs. The clinical manifestations of GD included hypermetabolic symptoms and lesions of the eyes, skin, and extremities [9]. GD can result in a high metabolic state and increased circulation that may cause tissue congestion. On the contrary, it can also lead to target tissue damage through inflammatory pathways.

The choroid is the vascular layer of the eye, and it can be scanned clearly by a relatively new technique, EDI-OCT. The choroidal morphology can provide information about some systemic diseases, such as diabetes [10], Alzheimer's disease [11], Parkinson's disease [12], and chronic kidney disease [13], and has been used to evaluate such diseases. The CT was obviously influenced by blood perfusion. For example, it becomes thicker when the patient's head was lowered [14]. The hallmark of GO is swelling of the extraocular muscles and increased orbital connective tissue and fat, which can cause an increase in intraorbital pressure [15]. This may lead to congestion and edema of the episclera and conjunctiva and affect the vascular drainage. The relative stagnation of venous blood on the episclera and conjunctive may be the major reason for the increasing CT.

Inflammation may be the other reason of changes in CT. Many inflammatory ocular diseases, including acute anterior uveitis [16], posterior scleritis [17], Behcet disease [18], and Vogt–Koyanagi–Harada syndrome [19], have been found to affect the congestion of choroidal tissues and result in changes in CT. It has been speculated that choroidal infiltration of inflammatory cells, increased exudation, increased vascular leakage, and ocular blood flow alternations may lead to changes in choroidal thickness [17]. As an autoimmune disease, many inflammatory factors, such as insulin-like growth factor-1 (IGF-1), can be found in GO [20]. These inflammatory factors may infiltrate the orbital fat and extraocular muscles [21], which is the adjacent structures of the outer surface of sclera. A similar mechanism with posterior scleritis may happen, and it may be the reason for changes in choroidal thickness. This may lead to apical orbital crowding and aggravate the stagnation of blood.

The association between GD and CT was not investigated until 2016. CT was found to be statistically increased in GO patients by Caliskan et al. [22] and Ozkan et al. [23]. These research results were partly similar to ours.

Our study design was more reasonable than the previous research. In addition to diseases, there are some other factors that may affect CT, including age, gender, and optical degree levels. CT has an age-related decline [24] and a sharper rise in men than in women [25]. The relationship between optical degree and CT is more obvious. CT has a negative correlation with spherical equivalent, which has been reported in numerous studies [24, 26]. Thus, age, gender, and optical degree should be consistent in a study of choroidal thickness. It was regretful that the optical degrees were not matched between the research and control groups in the studies of Caliskan and Ozkan, which may lead to the bias in the research of CT. In our study, these influential factors were rigidly matched in the two groups, which might have generated a more persuasive result. In the sample in Caliskan's study, all of the subjects had low optical degrees. CT was relatively thicker than in our study, and the difference was apparent at all the measured points.

A negative relationship between choroidal thickness and spherical equivalent had been reported in many articles [27]. In this research, the statistical relationship of diopter groups and central-nasal choroidal thickness is also found in control group but not found in the GO group. In control groups, refractive errors were the main affecting factor on choroidal thickness, but in the GO group, the Grave's disease is another critical influence which could neutralize the effect of refractive errors on choroidal thickness.

The results of our study also showed that there was no statistically significant relationship between CT and the degree of proptosis, which is inconsistent with the results of Dr. Caliskan's study. It is well-known that the exophthalmometric value is also affected by ocular diopters. Some research had showed that a significant negative relationship had found between the Hertal exophthalmometric measurements and spherical equivalent [28]. We did not restrict the ocular diopters of subjects in this study. In contrast, only low refractive errors patients were chosen in Caliskan's study, which may be the reason for the difference.

In our study, the CT in the GO group and the control group was thinner than those in previous studies. We think this may be due to racial differences. Previous studies mentioned that the CT of East Asians was thinner than that of European and American individuals [24].

The present study has several limitations. The first was the small sample size; thus, there might be a selection bias. Second, this was a cross-sectional study, and we did not evaluate changes after a follow-up period. In further studies, we will observe changes in CT in a long time, especially in the cases under a well control of thyroid function, which may help us to understand the underlying mechanism of increased CT in GO patients and to explore CT as a new evaluation index of GO.

In conclusion, in our study, CT was found to be significantly increased in GO patients when compared with the control group. However, the changes in CT were not associated with the CAS, degree of exophthalmos, or thyroid function testing.

## Figures and Tables

**Table 1:** Patients characteristics in GO and healthy control groups.

	GO group (*n* = 36)	Controls (*n* = 36)
Age (year)	39.8 ± 14.5	38.7 ± 14.5
Male (%)	25.0% (9/36)	25.0% (9/36)
Average spherical equivalent (D)		
** **Emmetropia level	0.48 ± 0.68	−0.18 ± 0.92
** **Low myopia level	−1.42 ± 0.51	−0.90 ± 0.97
** **Moderate myopia level	−4.45 ± 0.57	−3.95 ± 1.76
** **High myopia level	−7.45 ± 1.15	−6.25 ± 0.89

**Table 2:** CT (*μ*m) measured with EDI-OCT in the eyes of patients with GO and healthy controls.

CT	GO group (*n* = 36)	Controls (*n* = 36)	*t* value	*P*
Fovea	313.47 ± 100.32	256.33 ± 50.18	3.057	0.003^*∗*^
N1500	279.22 ± 85.80	223.14 ± 59.61	3.221	0.002^*∗*^
N3000	214.64 ± 75.52	176.69 ± 60.66	2.350	0.022^*∗*^
T1500	313.19 ± 80.36	250.92 ± 52.18	3.900	0.000^*∗*^
T3000	298.14 ± 82.75	239.47 ± 60.35	3.437	0.001^*∗*^

^*∗*^ *P* < 0.05.

**Table 3:** The relationship between choroidal thickness and refractive errors level (*F*: the statistical value of univariate analysis; *P*: probability value).

Group	T3000	T1500	SFCT	N1500	N3000
GO group	*F* = 0.293	*F* = 0.598	*F* = 1.141	*F* = 1.531	*F* = 2.295
	*P*=0.830	*P*=0.621	*P*=0.347	*P*=0.225	*P*=0.097

Controls	*F* = 1.979	*F* = 1.520	*F* = 3.678	*F* = 4.370	*F* = 4.169
	*P*=0.137	*P*=0.228	*P*=0.022	*P*=0.011	*P*=0.013

## Data Availability

The data used to support the findings of this study are available from the corresponding author upon request.
